# Genomic analysis of co-infection with *Wolbachia* and *Candidatus* Tisiphia in the sand fly *Sergentomyia squamirostris*

**DOI:** 10.3389/fmicb.2025.1577636

**Published:** 2025-05-09

**Authors:** Kentaro Itokawa, Akihiro Kuroki, Daisuke Kobayashi, Makoto Kuroda, Kyoko Sawabe, Haruhiko Isawa, Chizu Sanjoba

**Affiliations:** ^1^Department of Medical Entomology, National Institute of Infectious Diseases, Tokyo, Japan; ^2^Antimicrobial Resistance Research Center, National Institute of Infectious Diseases, Tokyo, Japan; ^3^Graduate School of Agricultural and Life Sciences, The University of Tokyo, Tokyo, Japan; ^4^Management Department of Biosafety, Laboratory Animal, and Pathogen Bank, National Institute of Infectious Diseases, Tokyo, Japan; ^5^Department of Medical Technology, Kumamoto Health Science University, Kumamoto, Japan

**Keywords:** sand fly, *Wolbachia*, *Candidatus tisiphia*, symbionts, genome

## Abstract

**Introduction:**

Endosymbiotic bacteria show diverse strategies to manipulate host reproduction for their survival in nature. The diversity of symbionts infecting hematophagous insects and their impact on host ecology could be crucial for developing effective control measures of disease-transmitting vectors. Sand flies are a group of small insects, with some species serving as vectors for leishmaniasis, bartonellosis, and arboviral diseases. *Sergentomyia squamirostris* is the only known species of sand flies found on the main islands of Japan. Although no medical implications exist for *S. squamirostris*, we conducted whole-genome sequencing to explore its potential relevance.

**Methods:**

We conducted whole-genome sequencing and *de novo* assembly of *S. squamirostris* using genomic DNA isolated from a single field-collected female insect sample. During this attempt, we incidentally obtained closed genomes of two new bacteria, wSSQ and RiSSQ, belonging to *Wolbachia* and *Candidatus* Tisiphia, respectively. We then investigated infection rates of both bacteria in two natural populations of *S. squamirostris* in Japan.

**Results:**

Phylogenetic analysis indicated that wSSQ and RiSSQ belonged to *Wolbachia* and *Ca*. Tisiphia, respectively. *Ca*. Tisiphia is also known as “Torix *Rickettsia*,” which is considered a large group of endosymbionts of invertebrates with no known pathogenicity to humans and animals. NGS read depths for both wSSQ and RiSSQ genomes were found to be high coverages, indicating that these bacteria are *S. squamirostris* endosymbionts. The infection rates of wSSQ and RiSSQ in the wild population of *S. squamirostris* varied in the two different localities in Japan, and co-infection with both bacteria was commonly seen. wSSQ was detected in both sexes of *S. squamirostris*, whereas RiSSQ was detected only in female sand flies.

**Conclusion:**

*Ca*. Tisiphia has recently been recognized as an underexplored endosymbiont with a widespread presence in invertebrates, including disease vectors. RiSSQ represents the first complete genomic information resource of *Ca*. Tisiphia infecting sand flies. Further research is needed to understand potential interactions between its host and other endosymbionts, as well as to explore the potential implications of disease control in the future.

## 1 Introduction

Phlebotominae sand flies (Diptera; Psychodidae) are a family of small insects with an adult body length of ~3 mm. Adult female sand flies obligately feed on the blood of vertebrates to facilitate oviposition. Male sand flies, on the other hand, do not have a hematophagous habitat and are believed to rely on plant sugars, such as nectar, as their energy source. Some species of sand flies are known to transmit significant human and animal diseases, such as leishmaniasis, arboviruses, and Bartonella. In Japan, only one species of the Phlebotominae insect, *Sergentomyia squamirostris*, had been described on the main island (Sanjoba et al., 2011) until *S. iriomotensis* was newly described in the Ryukyu Archipelago, just recently (Sanjoba and Miyagi, 2022). Although *S. squamirostris* may transmit some *Trypanosoma* parasites to amphibians (Feng and Chao, 1943), no evidence suggests that either species has medical implications for human or domestic animal health to date.

*Wolbachia* and *Rickettsia* belong to the order Rickettsiales, which comprises a large group of obligate intracellular parasitic bacteria. *Wolbachia*, belonging to the Ehrlichiaceae family, is a well-known maternally transmitted symbiont found in a wide range of arthropod species and some nematodes. In arthropods, *Wolbachia* shows intriguing phenotypes that manipulate host reproduction, including cytoplasmic incompatibility (CI), male killing (MK), feminization, and parthenogenesis (Hurst and Frost, 2015). All those phenotypes are considered to benefit the bacteria from propagating and maintaining within the host population. Additionally, certain strains of *Wolbachia* are known to establish obligate mutualistic relationships with their hosts, in which the bacteria provide essential nutrients, such as biotin (vitamin B7) (Foster et al., 2005; Hosokawa et al., 2010). CI is the most commonly observed host manipulation phenotype of *Wolbachia*, where offspring produced by infected males and uninfected females are inviable. Primary genetic factors of CI, *cifA*, and *cifB* (cytoplasmic incompatibility factors A and B) have recently been characterized (Beckmann et al., 2017; LePage et al., 2017). In the genomes of *Wolbachia, cifA* and *cifB* usually exist in pairs, with *cifA* located directly upstream of *cifB* (Martinez et al., 2021). Transgenic experiments have indicated that the expression of both *cifA* and *cifB* in paternal testes is required to induce CI death in embryos, whereas *cifA* expression alone in the maternal ovary can rescue this effect (Beckmann et al., 2017; LePage et al., 2017; Shropshire et al., 2018; Shropshire and Bordenstein, 2019). There is a relatively clear-cut relationship between the presence/absence of a functional *cifA*/*B* pair in the genome and that of the CI phenotype (Lindsey et al., 2018; Martinez et al., 2021). Certain strains of *Wolbachia* have been shown to suppress viral replication in transinfected mosquitoes (Walker et al., 2011). Due to this ability, along with their capacity to spread into uninfected populations autonomously as reproductive parasites as described above, *Wolbachia* has gained significant attention as a potential tool for disease control (Hoffmann et al., 2011; Caragata et al., 2021).

Rickettsiaceae includes important arthropod-borne human pathogens, such as some *Rickettsia* species causing spotted fever and typhus and *Orientia tsutsugamushi* causing scrub typhus. Although Rickettsiaceae were initially implicated as a group of such significant pathogens, the majority of known species in this family are now considered vertically transmitted symbionts of invertebrates and some protists (Werren et al., 1994; Lawson et al., 2001; Giorgini et al., 2010; Schrallhammer et al., 2013). “Torix group of *Rickettsia*” is also a group of Rickettsiaceae, which was first described in a species of leech, *Torix tagoi*, in 2005 (Kikuchi et al., 2002). Since its discovery, 16S gene fragments phylogenetically related to this bacterium have been detected in diverse species of arthropods (regardless of hematophagous habits) and some amoebae (Dyková et al., 2003). Neither an example of infection nor pathogenicity to vertebrates of this group of bacteria is known so far. Despite being found in a wide range of invertebrate species (Pilgrim et al., 2021), this group of bacteria has gained relatively little attention thus far, as the first draft (Pilgrim et al., 2017) and complete (Davison et al., 2022) genome assemblies of this group have only recently been published. Although they have been referred to as the “Torix group of *Rickettsia*” or the “Torix clade of *Rickettsia*,” Davison et al. (2022) have recently proposed a distinct genus, *Candidatus* Tisiphia, for these bacteria due to their apparent phylogenetic divergence from known members of the genus *Rickettsia*.

Numerous reports have described *Wolbachia* infection in wild populations of sand fly species (Cui et al., 1999; Ono et al., 2001; Rosário et al., 2022; Wang et al., 2022; Lozano-Sardaneta et al., 2023). Although fewer in number, some studies also suggest the presence of Rickettsiaceae endosymbionts in sand flies. Reeves et al. (2008) detected a 16S rRNA sequence in *Lutzomyia apache* in Wyoming, USA, that was 99% identical to a *Rickettsia* species previously reported from leeches. Li et al. (2016) detected a bacterial 16S rRNA homologous to *Rickettsia* from *Phelbotomus cinensis* in Sichuan, China. Lozano-Sardaneta et al. (2021) recently reported the detection of “Torix group” *Rickettsia* in *Psathyromyia aclydifera* in Los Tuxtlas, Mexico. These results suggest that infection of *Ca*. Tisiphia is not uncommon even among sand flies.

The small size and limited number of available established laboratory colonies make genomic analysis of this important group of insects challenging (Huang et al., 2024). Although *S. squamirostris* has not been recognized as a vector of any pathogen or nuisance pest in humans to date, we attempted to conduct a *de novo* genome assembly of a single specimen of wild-caught insects to test the technical feasibility of this approach and explore the potential medical implications of this species. During this attempt, we incidentally obtained two closed assemblies of the bacterial genome, each belonging to *Ca*. Tisiphia and *Wolbachia*, along with a moderately contiguous draft of the insect genome.

## 2 Materials and methods

### 2.1 Sample collection

**Figure 1:**
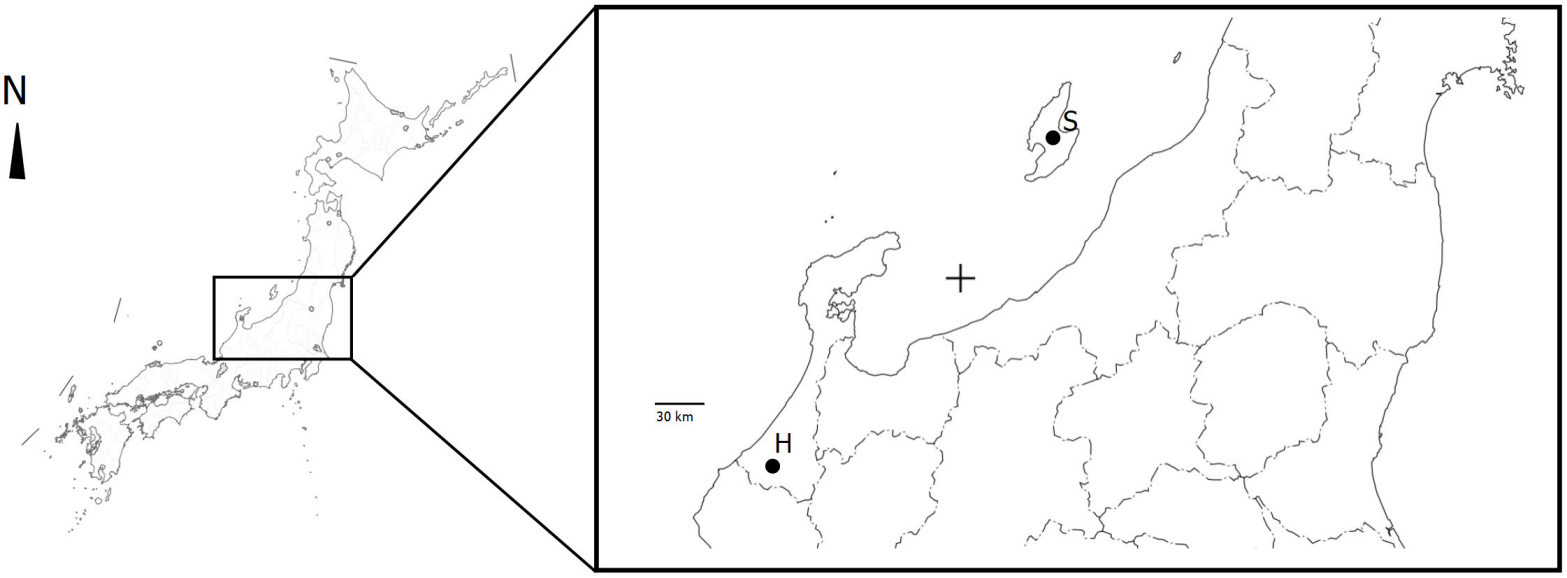
Map depicts the sample collection sites on the central-north coast of Japan. (S) Sado Island, Niigata and (H) Hakusan, Ishikawa Prefecture. The map was reproduced from the Geospatial Information Authority of Japan website (https://www.gsi.go.jp). This map is licensed under the Government of Japan Standard Terms of Use (Ver.2.0), which are compatible with the Creative Commons Attribution License 4.0 (CC By).

Adult sand flies, *S. squamirostris* were captured in Sado Island, Niigata prefecture (38°04′56″N 138°23′00″E) in 2018 and 2019 and Hakusan, Ishikawa prefecture, Japan (36°18′31″N 136°33′00″E) in 2021 (Figure 1) using CDC miniature light traps or sticky paper traps that were prepared using A4 paper coated with castor oil. The heads and abdomens of the collected sand flies were separated from the whole body and mounted on labeled slides containing Swan solution. The remainder of the body was preserved in 99.9% ethanol for further DNA extraction. Species were identified using published keys and descriptions based on the cibarium, pharynx, and spermathecae features in females, and genitalia, coxites, and styles in males (Lewis, 1987). The morphological characteristics were compared with previous descriptions (Newstead, 1923; Sanjoba et al., 2011) observed under BX51 and SZ61 microscopes (Olympus Co., Tokyo, Japan).

### 2.2 Genomic DNA extraction

The genomic DNA (gDNA) of the insects was purified individually using a MagExtract-genome kit (TOYOBO, Japan) in an 8-striped PCR tube. Purified gDNA was eluted in 25 μl of low-TE buffer (10 mM Tris–HCl, 0.1 mM EDTA, pH 8.0). The extracted DNA was quantified using the Qubit DNA HS kit (Thermo Fisher Scientific, the US).

### 2.3 Sequencing gDNA

The amount of gDNA obtained varied largely among individuals. One female insect from Sado Island in 2019 (Sado-F01) with the maximum recovery of gDNA (250 ng) was selected for sequencing analysis. A DNA library for nanopore sequencing was prepared from 180 ng gDNA from Sado-F01 with the SQK-LSK109 kit (Oxford Nanopore Technology, ONT, UK). The gDNA was end-prepped using the NEBNext Ultra II End Repair/dA-Tailing Module (New England Biolabs (NEB), US). The end-prepped DNA was purified with AMPureXP (Beckman Coulter Life Sciences, US) and ligated to the 1D adapter with Quick T4 DNA Ligase (NEB). The ligated DNA was purified using AMpure XP. A short fragment wash buffer (SFB) was used in the final bead-washing step. The library was sequenced in a single R.9.4.1 flow cell for MK1B (ONT). The obtained FAST5 files (raw signals) were basecalled with guppy v6.0.1 with the “super accuracy (sup)” model. Long reads obtained from nanopore sequencing were trimmed to 50-nt at both ends to remove adapter sequences using Seqtk (https://github.com/lh3/seqtk). Reads shorter than 1.5 kb were discarded for the subsequent assembly and polishing steps.

The PCR-free TruSeq library was prepared from 24 ng gDNA of Sado-F01 using the NEBNext Ultra II FS DNA Library Prep Kit for Illumina (NEB) with TruSeq DNA and RNA combinatorial dual-indexed adapters (Illumina, US). The library was sequenced using the NextSeq500 system (Illumina) with a 150PE mid-output kit along with other libraries. The obtained reads were trimmed and filtered using FASTP (Chen et al., 2018).

### 2.4 *De novo* assembly and QC

The trimmed long reads were subjected to *de novo* assembly by Flye v2.9 (Kolmogorov et al., 2019) with the “–meta” option (Kolmogorov et al., 2020). The obtained assembly was polished with medaka v1.5 (https://github.com/nanoporetech/medaka) using ONT long reads. The polished assemblies were further corrected with short reads using NextPolish v1.4.0 (Hu et al., 2020). Centrifuge v1.0.4 (Kim et al., 2016) was used to check for potential contamination of bacterial genomes in this initial assembly. Contigs of suspected symbiont genomes were further polished with short reads using PolyPolish software (Wick and Holt, 2022). From the insect genome, short contigs (smaller than 3,000 bp) and redundant haplotigs were purged with Purge_dups v1.2.5 (Guan et al., 2020) using information on short read coverage. Mitochondrial DNA was assembled from a subset (~130 Mb) of short reads using MEGAHIT v1.2.9 (Li et al., 2015). The completeness of the insect genome assembly was assessed by Benchmarking Universal Single-Copy Ortholog (BUSCO) v5.4.6 (Manni et al., 2021) against the diptera_odb10 dataset. For a comparative analysis, the available genome assembly and predicted protein sequences of *Lutzomyia longipalpis* ASM2433408v1 (accession no. GCF_024334085.1) (Huang et al., 2024), *Phlebotomus papatasi* Ppap_2.1 (GCF_024763615.1) (Huang et al., 2024), *P. argentipes* (GCF_947086385.1), and *P. perniciosus* asm_v2.0 (GCA_918844115.2) were benchmarked by the same version of BUSCO. The quality of the bacterial genomes was assessed with CheckM v1.2.2 (Parks et al., 2015) using the 2015_01_16 database. The mitochondrial genome was assembled from a subset (~130 Mb) of short reads using MEGAHIT v1.2.9 (Li et al., 2015).

### 2.5 Coverage analysis

*S. squamirostris* and bacterial genome assemblies were combined into a single FASTA file. Short read data were mapped to this combined reference by minimap2 (Li, 2018) with “-x sr -a” options, and then aligned reads were sorted by Samtools (Li et al., 2009). From the resulting BAM, Samtools depth with the “-a” option was used to obtain depth at every base position. The depth data were plotted on a histogram using Matplotlib (Hunter, 2007) in python3.

### 2.6 Gene annotation and comparative genomics of bacteria

The AutoMLST server (Alanjary et al., 2019) was used to classify the bacterial genomes into approximate taxonomic positions. Gene prediction and functional annotation were conducted using the DNA Data Bank of Japan (DDBJ) Fast Annotation and Submission Tool (DFAST) v1.2.18 (Tanizawa et al., 2018). Circos genome plots were generated using the GenoVi pipeline v0.2.16 (Krzywinski et al., 2009; Feldbauer et al., 2021; Cumsille et al., 2023). Bacterial genome assemblies belonging to *Wolbachia, Ca*. Tisiphia and closely related genomes for rooting trees were downloaded from the NCBI database (Supplementary Table S1). To infer the phylogenetic placement of RiSSQ and wSSQ among other Rickettsiaceae and Ehrlichiaceae, respectively, amino acid sequences of orthologs predicted by BUSCO v5.4.6 for the bacteria_odb10 lineage dataset were used to infer the phylogenies of bacteria. Among the 124 orthologs in bacteria_odb10, 54 and 70 orthologs (Supplementary Table S2) were selected as complete orthologs found in all genomes of Rickettsiaceae and Ehrlichiaceae, respectively, for comparison. Each ortholog family was multiple aligned with MAFFT v7.520 (Katoh and Standley, 2013) and trimmed by TrimAl v1.4.1 (Capella-Gutiérrez et al., 2009) with “–nogaps” mode. The trimmed alignments were concatenated into single alignments consisting of 12,415 and 19,882 amino acid residues for Rickettsiaceae and Ehrlichiaceae, respectively. The concatenated sequence was used for tree estimation using IQ-TREE v2.2.6 (Minh et al., 2020). Partitioning was implemented based on gene boundaries, and the best partition scheme and substitution models for each alignment were searched using the “-m MFP+MERGE” option (Lanfear et al., 2012; Kalyaanamoorthy et al., 2017). To estimate the branch support of the tree, 1,000 ultrafast bootstrap (UFBoot) resamplings were conducted (Hoang et al., 2018). For RiSSQ, the 16S RNA sequences were also compared with the 16S sequences of other *Ca*. Tisiphia (Supplementary Table S3), using MAFFT, TrimAl, and IQ-TREE. For metabolic analysis, gene functions were annotated with “anvi-run-kegg-kofams” using the KEGG Orthology database (last updated: 24 November 2020), and the completeness of metabolic pathways was estimated with “anvi-estimate-metabolism” in Anvi'o v7.1 (Aramaki et al., 2020; Eren et al., 2021; Veseli et al., 2023).

### 2.7 Search for the prophage region and *cifA* and *cifB* homologs

Prophage sequences were identified using PHASTEST (Wishart et al., 2023) (accessed 2 June 2024). The dataset of Martinez et al. (2021) was used as a reference sequence for the well-characterized cifA and cifB. The BLASTP algorithm (Altschul et al., 1990) was used to search for similar sequences, using the references as targets and the predicted protein sequences of wSSQ or RiSSQ as queries. Matches to the cifB references were restricted to the first 500 amino acids from their N-terminal because this region contains highly conserved domains (AAA-ATPase-like and PD-(D/E)XK nuclease domains) among cifBs, whereas the rest of the region contains domains such as ankyrin repeats, which tend to result in spurious hits to non-cif proteins. We also generated profile hidden Markov models (hmms) from multiple sequence alignments of each reference cif protein using “hmmbuild,” and used them to search for homologs in predicted proteins of wSSQ or RiSSQ by using “hmmsearch” in the HMMER v3.1b2 package (Eddy, 2011).

### 2.8 Repeat masking, gene prediction, and annotation of *S. squamirostris* genome

Repeat elements in *S. squamirostris* genome assembly were predicted using RepeatModeler v2.0.5 (Flynn et al., 2020) with the “-LTRStruct” option. The assembly was then masked by RepeatMasker v4.1.5 (Tarailo-Graovac and Chen, 2009) using predicted repetitive sequence families. The BRAKER3 pipeline (Lomsadze et al., 2005; Stanke et al., 2006, 2008; Gotoh, 2008; Iwata and Gotoh, 2012; Buchfink et al., 2015; Hoff et al., 2016, 2019; Bruna et al., 2020a,b) was used to predict protein-coding sequences. The combined protein sequences of *Phlebotomus papatasi* (GCF_024763615.1) and *Lutzomyia longipalpis* (GCF_024334085.1) were used as reference databases. Orthomapper v3.0.5 was used to cluster the predicted proteins with known dipteran orthologous groups using OrthoDB v11 (Kuznetsov et al., 2023). Barrnap v0.9 (https://github.com/tseemann/barrnap) was used to predict ribosomal RNA (rRNA) genes, and tRNAscan-SE v2.0.12 (Chan et al., 2021) was used to predict transfer RNA (tRNA) genes. MitoZ v3.6 was used to predict and annotate mtDNA genes (Meng et al., 2019).

### 2.9 Insect phylogenomics

Protein sequences of single-copy orthologs extracted from the results of the BUSCO analysis (insecta_odb10) were used for the phylogenomic analysis of insects. The compared taxons included three mosquito genomes, *Aedes aegypti* AaegL5.0 (GCA_002204515.1), *Anopheles gambiae* AgamP3 (GCA_000005575.1), and *Culex quinquefasciatus* CulPip1.0 (GCA_000209185.1); one moth fly genome, *Clogmia albipunctata* ASM101494v1 (GCA_001014945.1); and four sand fly genomes, *L. longipalpis* ASM2433408v1 (GCF_024334085.1), *P. argentipes* (GCF_947086385.1), *P. papatasi* Ppap_2.1 (GCF_024763615.1), and *P. perniciosus* (GCA_918844115.2). Among the 1,367 insecta_odb10 ortholog groups, 1,004 were selected as complete genes among all species. Each ortholog group was multi-aligned by MAFFT v7.520 (Katoh and Standley, 2013), and the alignments were trimmed by TrimAl v1.4.1 (Capella-Gutiérrez et al., 2009) with “–nogaps” mode. The trimmed alignments were concatenated using SeqKit v2.6.1 (Shen et al., 2016), which included 355,011 amino acid residues per species. The concatenated sequence was used for tree estimation using IQ-TREE v2.2.6 (Minh et al., 2020) with 1,000 UFBoot resampling (Hoang et al., 2018). Partitioning was implemented based on gene boundaries, and the best partition scheme and substitution model search were performed with the “-m MFP+MERGE” option. Individual gene trees were also constructed from each trimmed alignment using IQ-TREE with the “-m MFP” option. Internal branches with low support values (UFBoot < 10%) in each tree were contracted to give multifurcation. The coalescent phylogenetic tree was then estimated by Astral v5.7.8 (Zhang et al., 2018) using contracted individual gene trees.

### 2.10 Population infection rate analysis

The presence of RiSSQ and wSSQ in other fields collected *S. squamirostris* samples was checked by PCR using specific primers (with the M13F tail on the forward primers) targeting the genes *OmpA* (RiSSQ chromosome), *DnaA* (RiSSQ plasmid), and *wsp* (wSSQ chromosome; Table 1). As a positive control to ensure the quality of the extracted gDNA, a fragment of the *NAD5* gene in *S. squamirostris* mitochondria was also amplified. The PCR mixture (10 μl) consisted of 1x PCR Buffer for KOD FX (TOYOBO), 0.4 mM dNTPs, 0.25 μM of each primer, 0.02 U/μl of KOD-FX polymerase (TOYOBO), and 0.5–2 ng of gDNA, and the thermal condition was 95°C for 15 s followed by 40 cycles of 95°C for 15 s, 57°C for 15 s, and 68°C for 45 s. Each PCR product was visualized on 1.5% agarose gel containing GelRed dye (Biotium, US). DNA was purified from positive samples using AMPureXP (× 1.0) and subjected to capillary sequencing (GeneWiz, Japan) using the M13F primer.

**Table 1:** Primers used to detect symbionts and host mitochondria.

**Primer**	**Sequence**	**References**	**Approx. product size (kb)**
wsp_F1	[M13F] + GTCCAATARSTGATGARGAAAC	Baldo et al., 2006	0.6
wsp_R1	CYGCACCAAYAGYRCTRTAAA	Baldo et al., 2006	
RiSSQ_ompA_F1	[M13F] + CGTCTGCTGATGTGCCTACA	This study	0.9
RiSSQ_ompA_R1	AGTCGTCACGCTGTTAGCAA	This study	
RiSSQ_DnaA_F1	[M13F] + ACTCCATACCCTACCTGCGT	This study	0.9
RiSSQ_DnaA_R1	GTTTTGGCGGTGCTTTTTGC	This study	
SSQ_NAD5_F1	[M13F] + TAGCTGCCCCAACTCCTGTA	This study	0.7
SSQ_NAD5_R1	GGGGCAGGGAATAATAGCCA	This study	
M13F	GTAAAACGACGGCCAGT		

### 2.11 Statistical analysis

Fisher's exact test was conducted in R version 4.2.2 (R Development Core Team, 2014) using the fisher.test() function.

## 3 Results

### 3.1 Two bacterial genomes assembled from an individual insect

Genomic DNA (gDNA) was extracted individually from *S. squamirostris* collected from Sado Island, Japan, in 2018 and 2019 and from Ishikawa Prefecture in 2021. We obtained 5.2 G base of long reads (N50 16.6 kb) by ONT and 15 Gbase of 150 bp paired-end short reads by Illumina. After *de novo* assembly, polishing, and correction, bacterial contamination in the resulting assembly was checked by centrifuge (Kim et al., 2016). This quality check indicated that the initial assembly contained two large contigs of potential bacterial genomes in addition to the lambda phage DNA sequence, which was used for quality control (DNA CS) in the Nanopore library preparation kit. AutoMLST (Alanjary et al., 2019) phylogenomic analysis placed one large bacterial contig (RiSSQ) close to the genome of RiCNE (GenBank accession number GCF_002259525.1) (Pilgrim et al., 2017), a member of *Ca. Tisiphia* in a biting midge, *Culicoides newsteadi*. Another large bacterial contig (wSSQ) was assigned to the *Wolbachia* clade.

### 3.2 Genome of RiSSQ

**Figure 2:**
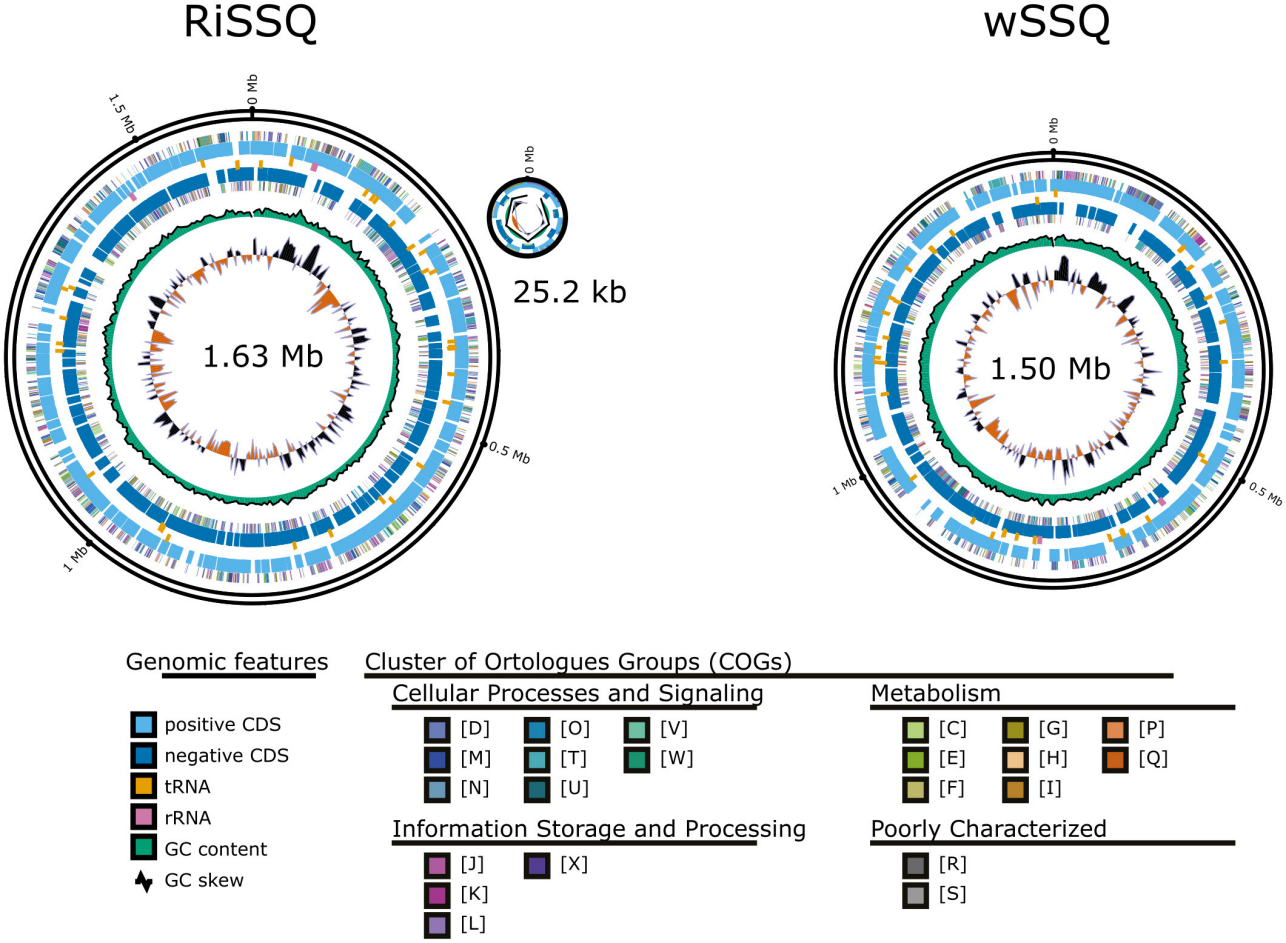
GenoVi circular representation of RiSSQ (+plasmid) and wSSQ. Genome size and CheckM score are described for each genome. The alphabet symbols for each COG category correspond to the following; D: cell cycle control, division, chromosome partitioning; M: cell wall/membrane/envelope biogenesis; N: cell motility; O: post-translational modification, protein turnover, chaperones; T: signal transduction mechanism; U: intracellular trafficking, secretion, and vesicular transport; V: defense mechanism; W: extracellular structures; Y: nuclear structure; Z: cytoskeleton; A: RNA processing and modification; B: chromatin structure and dynamics; J: translation, ribosomal structure, and biogenesis; K: transcription; L: replication, recombination, and repair; X: mobilome: prophages, transposons; C: energy production and conversion; E: amino acid transport and metabolism; F: nucleotide transport and metabolism; G: carbohydrate transport and metabolism; H: coenzyme transport and metabolism; I: lipid transport and metabolism; P: inorganic ion transport and metabolism; Q: secondary metabolites biosynthesis, transport, and metabolism; R: general function prediction only; and S: function unknown.

**Figure 3:**
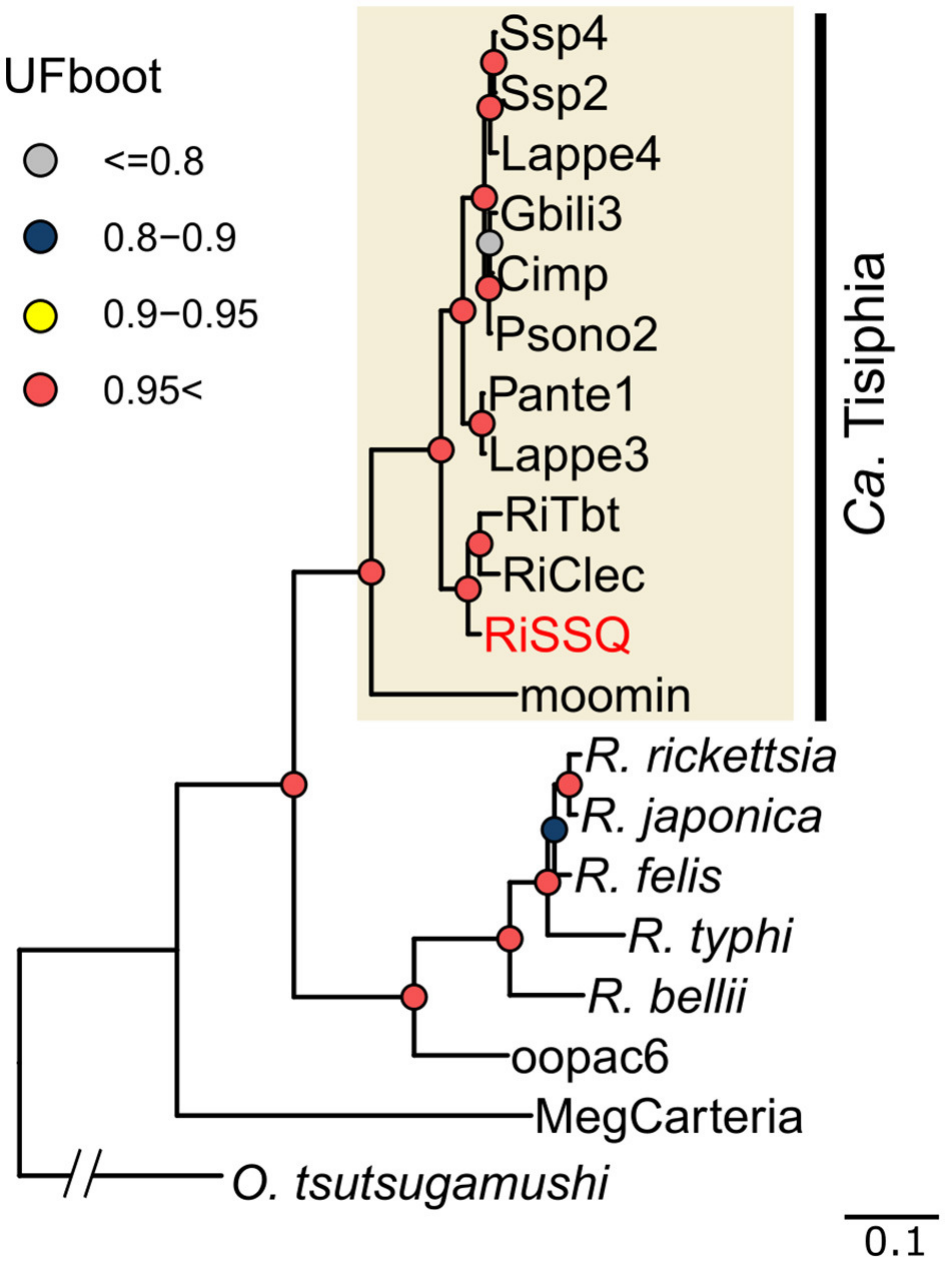
Phylogenetic placement of RiSSQ in *Ca*. Tisiphia and other Rickettsiaceae based on concatenated protein sequences of 54 orthologs. The details of each taxon written in the alias, and genes used for phylogenetic analysis are described in Supplementary Tables S1, S2, respectively.

The closed assembly of the RiSSQ chromosome (1,627,389 bp, 32.8 GC%; accession no. GCA_042848025.1) contained 1,541 coding sequences (CDSs), 35 tRNA genes, and 3 rRNA genes (Figure 2). In addition to the chromosome, we found a circular small contig (25,235 bp, 31.04 GC%) of a putative RiSSQ plasmid containing 31 CDSs, one of which has homology to chromosomal replication initiator proteins (DnaA), LF885_07200 (UCM86407, 59% amino acid identity) and LF885_07390 (UCM86444, 57% amino acid identity), encoded in the two plasmids in RiCimp, a strain of *Ca*. Tisiphia found in *Culicoides impunctatus* (Davison et al., 2022). The CheckM analysis scored the quality of genome assembly with 99.05% completeness and 1.66% contamination. The metabolic pathways predicted by the RiSSQ gene set included the complete pentose phosphate pathway (PPP) (Supplementary Figure S1), which is consistent with other members of *Ca*. Tisiphia (Pilgrim et al., 2017; Davison et al., 2022). On the other hand, the gene set for the biotin synthesis pathway, which is often associated with mutualistic symbionts (Nikoh et al., 2014), was not present in the RiSSQ genome (Supplementary Figure S1). Furthermore, no homologs of *cifA*/*B* were found. Phylogenetic analysis using 54 complete orthologous protein sequences placed RiSSQ within *Ca*. Tisiphia clade (Figure 3). Among *Ca*. Tisiphia and RiSSQ genomes clustered with the RiClec and RiTbt genomes found in *Cimex lectularius* and *Bemisia tabaci*, respectively, which has been described as “Leech subclade” in Davison et al. (2022). Some of the *Ca*. Tisiphia 16S rRNA sequences were retrieved from the NCBI nucleotide collection (accessed in January 2024). Although 16S rRNA sequences did not have enough power to resolve detailed phylogenetic structures, the 16S gene sequences of “*Rickettsia*” in other sand fly species, *Phlebotomus chinensis* in Henan, China, 2015 (Li et al., 2016) (KX363666.1 and KX363668.1), *Lutzomyia apache* collected in Wyoming, USA (Reeves et al., 2008) (EU223247.1), and *Psathyromyia aclydifera* collected in Los Tuxtlas, Mexico (Lozano-Sardaneta et al., 2021) (MT158807.1) all belong to the *Ca*. Tisiphia clade (Supplementary Figure S2). The coverage of mapped NGS short reads was calculated to estimate the relative abundance of each bacterial chromosome and plasmid within a female *S. squamirostris* (Sado-F01) whole body. A single peak in the read depth distribution was observed for each haplotig-purged insect chromosome, mitochondria, bacterial chromosome, and plasmid (Supplementary Figure S3). The RiSSQ chromosome and plasmid had ~10.6- and 41.8-fold more read depths, respectively, than the insect chromosomes.

### 3.3 Genome of wSSQ

**Figure 4:**
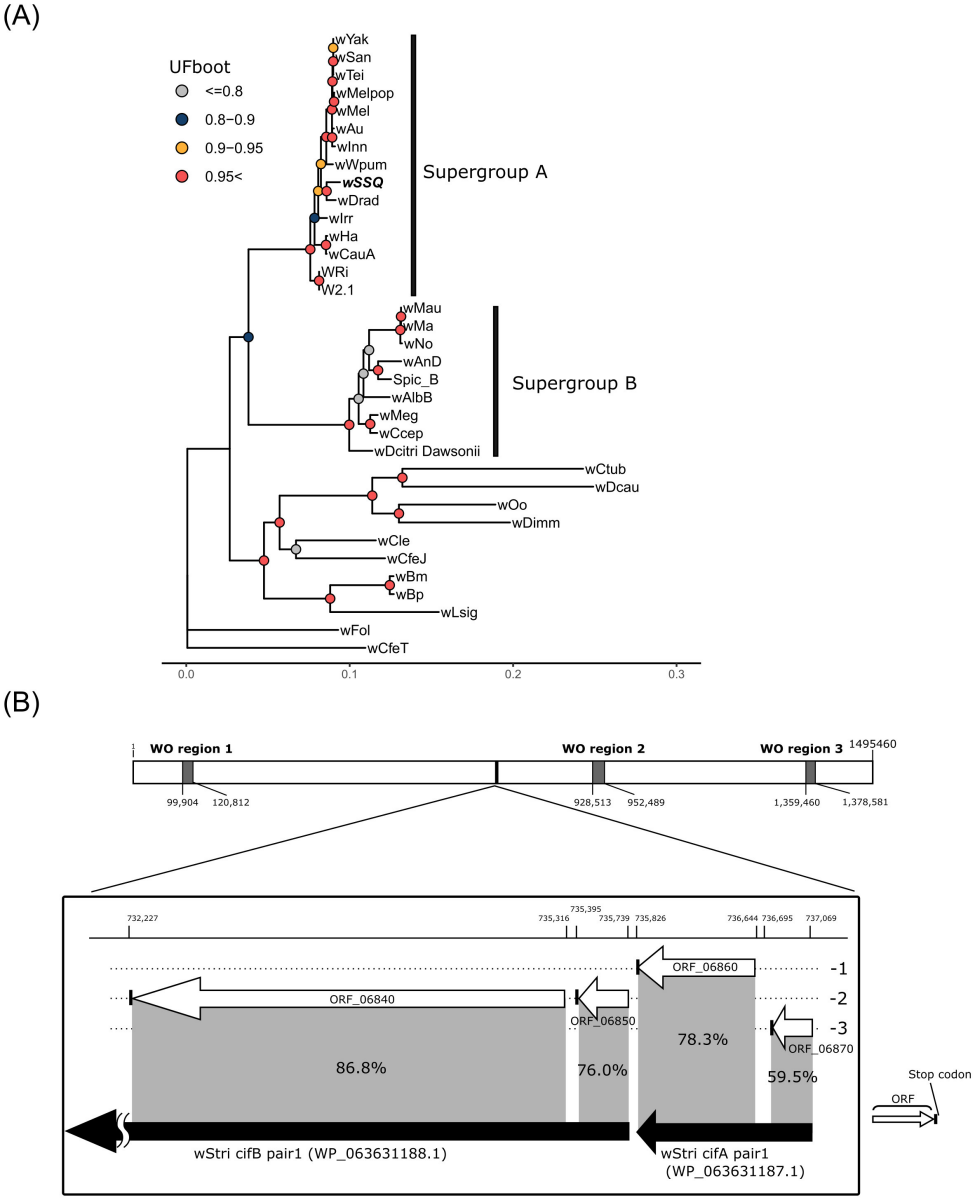
**(A)** Phylogenetic placement of the wSSQ genome among *Wolbachia* genomes based on concatenated protein sequences of 54 orthologs. The *Anaplasma marginale* (ASM2030v1) genome was used to root the tree. The details of each taxon written in the alias and genes used for phylogenetic analysis are described in Supplementary Tables S1, S3, respectively. **(B)** Structure of ORF_06840-70 in the wSSQ genome. Levels of the dotted line indicate the translational frame in both strands. White box arrows indicate open reading frames (ORFs), and vertical lines at the tip of ORFs indicate stop codons. Black box arrows show the wStri cifA and cifB protein sequences aligned to each wSSQ ORF with BLASTP hit %identity in the gray shade.

The chromosome of wSSQ (1,495,460 bp, 34.9 GC%, accession no. GCA_042853495.1) contained 1,376 CDSs, 35 tRNA genes, and three rRNA genes (Figure 2). CheckM analysis scored the quality of genome assembly with 99.79% completeness and 0.21% contamination. Phylogenomic analysis of other *Wolbachia* genomes, outgrouped by *Anaplasma marginale*, using 70 orthologous protein sequences, indicated that wSSQ belongs to the Supergroup A clade of *Wolbachia* (Figure 4A). The relative depth of mapped NGS short reads on the wSSQ chromosome was 3.9-fold higher than that on insect chromosomes and approximately three-fold lower than that of RiSSQ (Supplementary Figure S3). The gene set for the biotin synthesis pathway is absent in the wSSQ genome (Supplementary Figure S1).

Four open reading frames (ORFs; ORF_06840, ORF_06850, ORF_06860, and ORF_06870) were homologous to known *cifA* or *cifB* (Martinez et al., 2021) detected by both BLASTP and HMMER searches. These ORFs were located consecutively and directly (Figure 4B) outside the predicted WO prophage regions of the wSSQ genome. The results of BLASTP alignment indicated that ORF_06840 and ORF_06850 represented fragments of a single *cifB* gene that had been split by a premature stop codon. This mutation separated the “AAA-ATPase-like domain” on the N-terminal from other parts of the original cifB protein. ORF_06860 and ORF_06870 were also considered to represent fragments of a single *cifA* gene, which has been separated due to a potential frameshift mutation (Figure 4B). The tentative wSSQ cifA (ignoring the stop codon) and cifB (joining two fragmented ORFs) amino acid sequences have high homology to cifA pair1 in wStri and cifB single 2 in *Wolbachia* in *Acromyrmex echinatior*, both of which belong to the type V group, according to Martinez et al. (2021) (Supplementary Figure S4).

### 3.4 Genome of *S. squamirostris*

**Figure 5:**
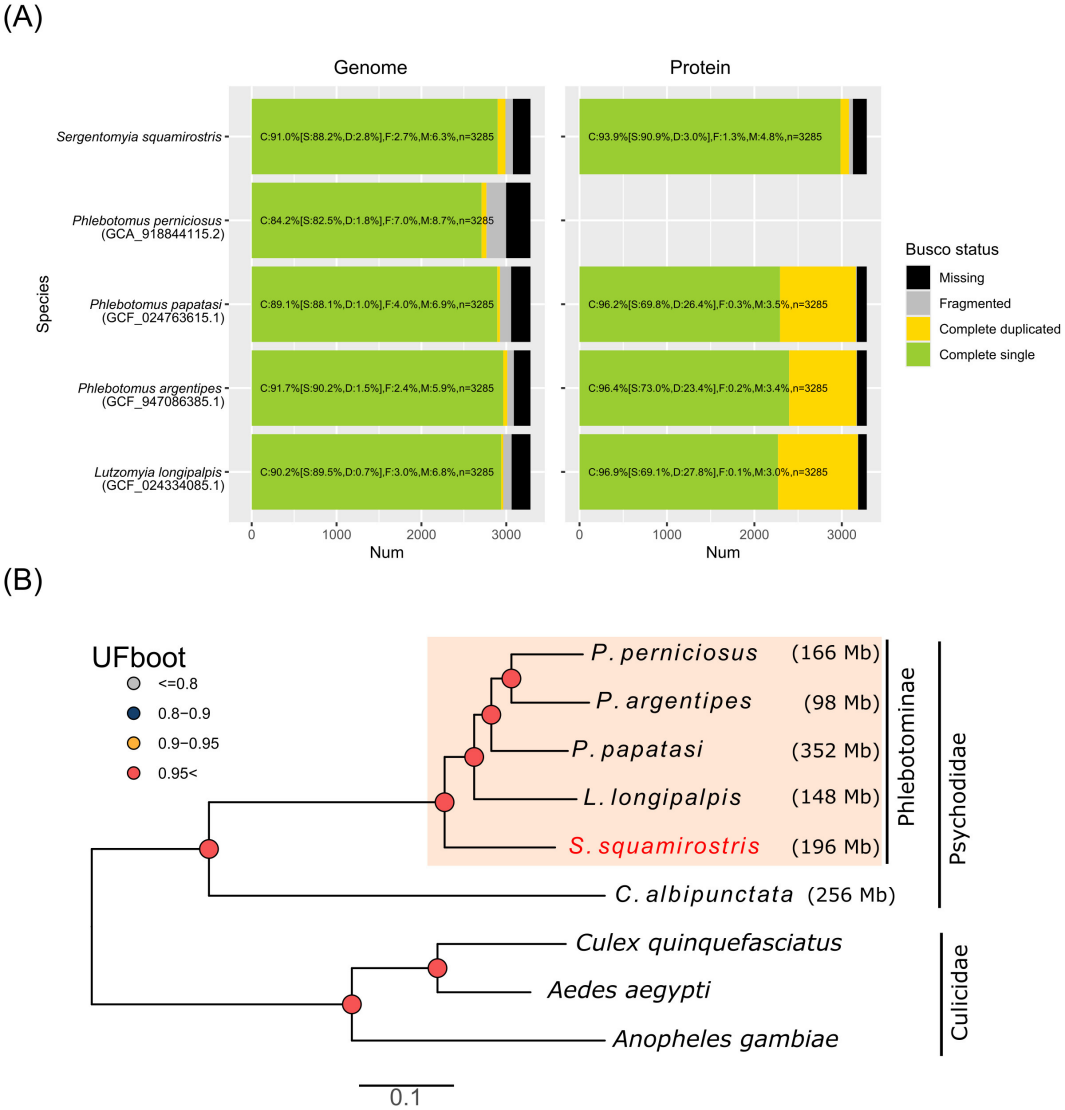
**(A)** Comparison of BUSCO scores for assembly and protein prediction of *S. squamirostris* to those in the reference genomes of sand fly species. Protein data for *P. perniciosus* were not registered as of January 2024. **(B)** ML phylogenetic tree for sand fly genomes based on concatenated protein sequences of 1,004 orthologs (Supplementary Table S4). Genomes of the drain fly (*Clogmia albipunctata*) and three mosquito (Culicidae) species are included in the root of the tree.

The obtained assembly for the haploid genome of *S. squamirostris* (Ssqu_1.0, accession no. GCA_042850755.1) consisted of 2,379 contigs for chromosomal DNA (total = 195,863,774 bp, N50 = 215 kb, GC% = 35.58) and one contig for the complete mitochondrial DNA sequence (total = 15,381 bp, GC% = 23.1). BUSCO v5.4.6 (diptera_odb10, 3,285 ortholog groups) analysis found that 91.0% were completely conserved orthologs with 2.8% duplication, which was comparable to the available assemblies for other sand fly species (Figure 5A). BRAKER3 pipeline using the reference protein sequences of *P. papatasi* and *L. longipalpis* predicted 15,013 protein-coding genes. The predicted set of proteins captured 93.9% of diptera_odb10 orthologs (Figure 5A). Phylogenomic analysis was conducted using 1,004 concatenated protein orthologs. The inferred tree, rooted in the mosquito and moth fly (Psychodidae) genomes, placed *S. squamirostris* in the basal clade of the Phlebotominae family (Figure 5B). Although this tree topology was supported in the coalescent species tree estimated by ASTRAL using individual gene trees, there was a relatively high discordance of topologies among the individual gene trees, as seen in low quartet support scores (46.0–94.41) for internal branches within Phlebotominae (Supplementary Figure S5).

### 3.5 Population analysis

**Figure 6:**
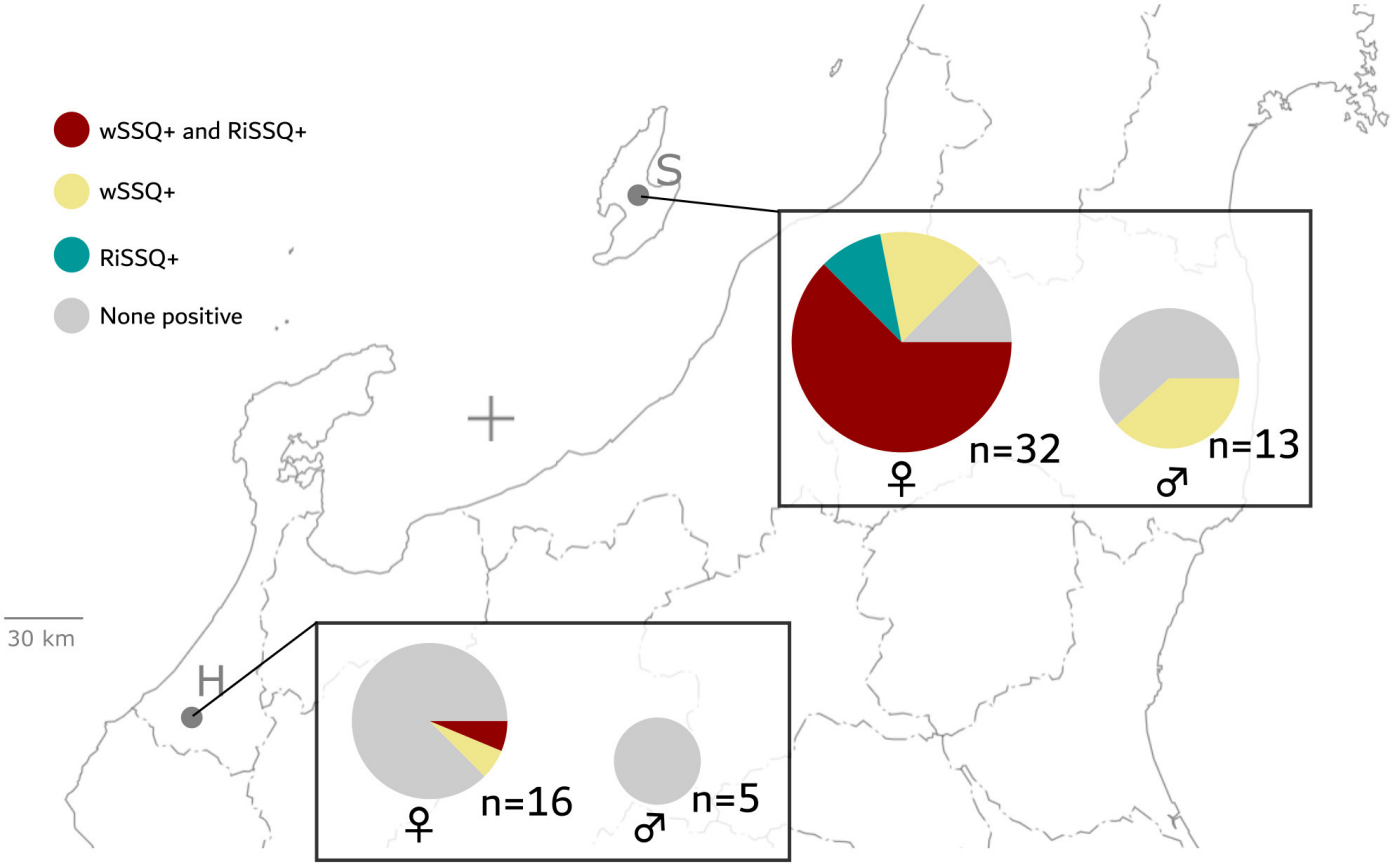
Frequencies of RiSSQ and/or wSSQ infection statuses by sex and population (S: Sado Island, Niigata prefecture or H: Hakusan, Ishikawa Prefecture) in *S. squamirostris*, based on the results described in Table 2. The background map corresponds to the one in Figure 1.

The genomic DNA of an additional 31 females and 13 males collected during 2018–2019 from Sado Island was tested for infection with RiSSQ and wSSQ using PCR (Figure 6 and Table 2). *S. squamirostris* mitochondrial *NAD5* gene was used as a positive control to assess the integrity of the extracted DNA. Among the 32 female *S. squamirostris* (including Sado19-F01) samples during this period, 23 (72%) flies were PCR positive for RiSSQ gene markers, and 24 (75%) were positive for wSSQ chromosome gene markers. In contrast, among the 13 males investigated, 5 (38%) flies were positive for the wSSQ marker, suggesting a lower infection rate than females (*p* = 0.016 in Fisher's exact test for female-vs.-male positive rate), and were all negative for RiSSQ (*p* = 6.8 × 10^−8^ in Fisher's exact test for female-vs.-male positive rate). The RiSSQ plasmid marker (*DnaA*) was completely associated with the RiSSQ chromosome marker. Among the 32 females, 20 individuals were co-infected with both RiSSQ and wSSQ (*p* = 0.076 in Fisher's exact test under the “independent infection” null hypothesis). We also analyzed 16 females and six males of *S. squamirostris* in Ishikawa Prefecture, ~200 km across the strait from Sado Island (Figure 6 and Table 2). One male specimen was excluded from the analysis because it tested negative for *NAD5* (positive control). In the Ishikawa population, only one female was positive for RiSSQ, and two females were positive for wSSQ out of the 15 NAD5 positive specimens. This RiSSQ-infected female was also infected with wSSQ. The infection rates of RiSSQ and wSSQ among the female population were significantly different between Sado and Ishikawa (*p* = 2.8 × 10^−5^ and 1.0 × 10^−4^, respectively, in the Fisher's exact test). Although the amplified gene markers (*NAD5, OmpA, DnaA*, and *wsp* genes) of the symbionts were directly sequenced and compared to each assembled reference of RiSSQ and wSSQ from Sado-F01 individuals, no variable nucleotide site was detected.

**Table 2:** Infection of RiSSQ and wSSQ in two wild populations of *S. squamirostris* in Japan.

**Location**	**Sex**	**RiSSQ chromosome (*OmpA*)**	**RiSSQ plasmid (*DnaA*)**	**wSSQ chromosome (*Wsp*)**	**Num**.
Sado Island	F	**+**	**+**	**+**	20
	F	–	–	+	5
	F	+	+	–	3
	F	–	–	–	4
	M	+	+	+	0
	M	–	–	+	5
	M	+	+	–	0
	M	–	–	–	8
Ishikawa	F	+	+	+	1
	F	–	–	+	1
	F	+	+	–	0
	F	–	–	–	14
	M	+	+	+	0
	M	–	–	+	0
	M	+	+	–	0
	M	–	–	–	4

F, female; M, male, +, positive; –, negative.

## 4 Discussion

In this study, we assembled the closed genomes of two bacteria, RiSSQ and wSSQ, from gDNA obtained from wild-caught *S. squamirostris*. Both bacteria belong to large taxonomic clades consisting of numerous known endosymbionts of arthropods. RiSSQ is a member of an uncultured group of *Rickettsiaceae* known as the “Torix group of *Rickettsia*.” Based on a recent phylogenomic analysis, Davison et al. (2022) admitted apparent distinctness from members of the genus *Rickettsia* and proposed a new genus “*Candidatus* Tisiphia,” for this group. The high coverage of the NGS reads of the RiSSQ chromosome and the plasmid, which has helped to assemble the complete genome, indicates a high abundance of this bacteria within an adult female of *S. squamirostris*. A complete gene set of PPP was found in the RiSSQ genome, which is a distinct metabolic feature of *Ca*. Tisiphia and *Ca*. Megaira from the other members of Rickettsiaceae (Pilgrim et al., 2017). Davison et al. (2022) hypothesized that the PPP is an ancestral feature of the main *Rickettsia* clade, which has been lost in several lineages. RiSSQ has at least one plasmid that is found in all RiSSQ-positive insects in the wild population of *S. squamirostris*. *Ca*. Tisiphia in *Culicoides impunctatus* (RiCimp) contains two plasmids (Davison et al., 2022). Lehman et al. (2024) recently reported that the plasmid pRiCimp001 in RiCimp carries a unique RalF effector of the T4SS. However, this characteristic may not be present in RiSSQ, as no *RalF* homolog was found in either its plasmid or chromosome. wSSQ belongs to *Wolbachia* supergroup A, which includes strains associated with CI, MK, and parthenogenesis induction. Although the molecular basis of these reproductive phenotypes has not been fully elucidated, *cifA* and *cifB* are considered essential factors for CI in *Wolbachia*. It is assumed that wSSQ may have lost its CI-inducing ability due to functionally disruptive mutations in the *cifA* and *cifB* homologs. Horizontal acquisition and pseudogenization of *cif* genes are frequently seen in *Wolbachia* (Martinez et al., 2021). However, it should be noted that Owashi et al. (2024) have recently reported that a *Rickettsia* strain with only split *cifB* induces CI in Hemipteran insect, *Nesidiocoris tenuis*. Thus, crossing experiments are needed to determine the presence or absence of CI in *S. squamirostris* with wSSQ.

One notable feature of RiSSQ is its high abundance in adult female sand flies. This raises an intriguing question of whether *Ca*. Tisiphia could interfere with other symbionts, such as *Wolbachia*, or pathogens co-infecting the same insect. Such potential interaction could have important medical implications for arthropod-borne diseases. However, further investigation is required because our knowledge of this group of bacteria is quite limited. In particular, the modes of transmission and reproductive phenotypes are still unknown for the majority of *Ca*. Tisiphia members, including RiSSQ. The maternal transmission of *Ca*. Tisiphia in arthropod hosts has been confirmed, either directly or indirectly, in several cases (Watanabe et al., 2014; Pilgrim et al., 2020; Thongprem et al., 2020). However, maternal transmission alone does not fully account for the maintenance and propagation of symbionts within the host population (Fine, 1975). For this reason, *Wolbachia* and other endosymbionts that mainly rely on maternal transmission must exhibit their unique phenotypes (either mutualism or reproductive parasitism) to survive in nature. Although *Ca*. Tisiphia exhibits both paternal and maternal transmission in leafhoppers (Watanabe et al., 2014), the transmission dynamics in *S. squamirostris* remain unknown. The absence of RiSSQ in adult males also suggests that CI may not be occurring, while other reproductive manipulations such as MK, thelytokous parthenogenesis, or feminization are possible. On the other hand, wSSQ was detected in both male and female hosts. Interestingly, the wSSQ strain also showed that the infection rate was moderately biased to females in the Sado population. It was reported that a *Wolbachia* strain in *Aedes albopictus*, wAlbA, showed different density dynamics between females and males, where the infection rapidly becomes undetectable in male adults (Tortosa et al., 2010). Alternatively, such a pattern of moderate bias in infection rate between the sexes could have resulted from a conflict between a sex-distortion (MK, parthenogenesis, or feminization) effect of symbionts and a suppressor gene evolving in the hosts (Hornett et al., 2022). Nevertheless, we will need reliable sex markers or an established colony under laboratory conditions to test these hypotheses.

The draft genome of *S. squamirostris* showed BUSCO completeness comparable to that of other available genome assemblies of sand fly species. This suggests that obtaining reference-quality assemblies using a wild-caught single insect and a portable nanopore sequencer is a feasible objective. Phylogenetic analysis using >1,000 protein sequences of single-copy orthologs placed *S. squamirostris* in the basal lineage of Phlebotominae. This result aligns with a recent study by Charamis et al. (2024), which placed *Sergentomyia schwetzi* as the basal lineage of Phlebotominae based on alignments with 575 single-copy orthologs obtained from transcriptomic analysis. On the other hand, studies using a few gene markers, such as those by Grace-Lema et al. (2015), supported different phylogenetic placements. Expanding the availability of reference genomes for more species within this group of insects will significantly enhance our understanding of their evolution.

## 5 Conclusion

*S. squamirostris* is naturally co-infected with *Ca*. Tisiphia and *Wolbachia*, offering insights into host–endosymbiont interactions. However, the potential influence of these bacteria on pathogen transmission in sand flies still remains unclear. Further research is required to understand their impact on host ecology and potential implications for disease control.

## Data Availability

Genome assemblies, predicted genes, and annotations of *S. squamirostris* (GCA042850755.1), RiSSQ (GCA042848025.1), and wSSQ (GCA_042853495.1) were registered in DDBJ and are available through the International Nucleotide Sequence Database Collaboration (INSDC) network under BioProject PRJDB17301. Raw NGS read sequences from the MinION and NextSeq500 analyses are also available under accession no. DRX508768 and DRX508769, respectively.
