# Supplementary material for: Genomic analysis of co-infection with Wolbachia and Candidatus Tisiphia in the sand fly Sergentomyia squamirostris
Source: Front Microbiol. 2025 May 9;16:1577636. doi: 10.3389/fmicb.2025.1577636 (PMC12123380; doi:10.3389/fmicb.2025.1577636)
Supplement: Supplementary file 5 [file Image_1.pdf]

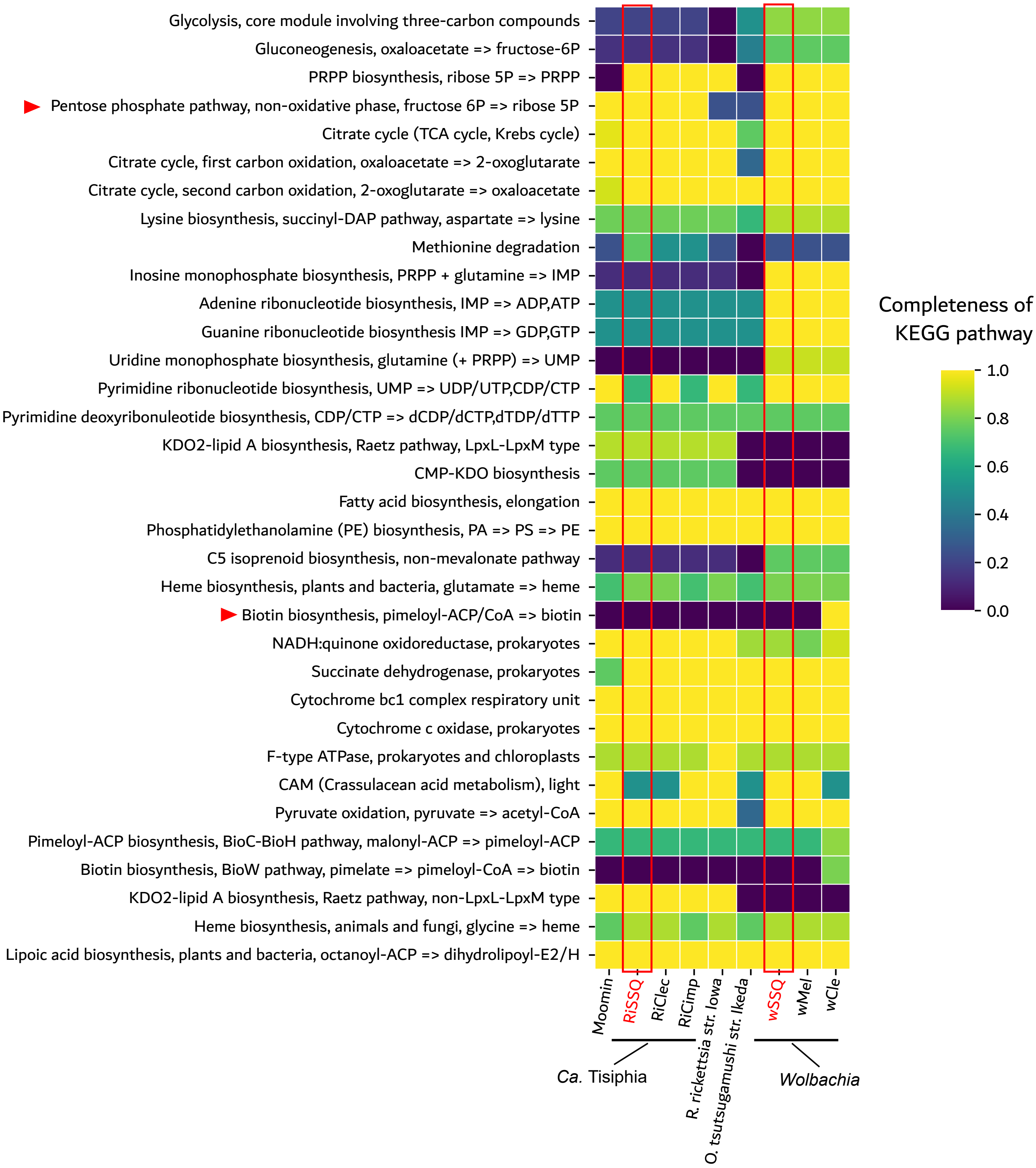

Figure S1 Predicted KEGG pathway completion for RiSSQ, other *Ca*. Tisiphia, *Rickettsia rickettsia*, and *Orientia tsutsugamushi*. Only modules with completeness ≥ 0.75 in either genome are displayed. Red arrowheads indicate the pentose phosphate pathway (PPP) and biotin biosynthesis pathway.

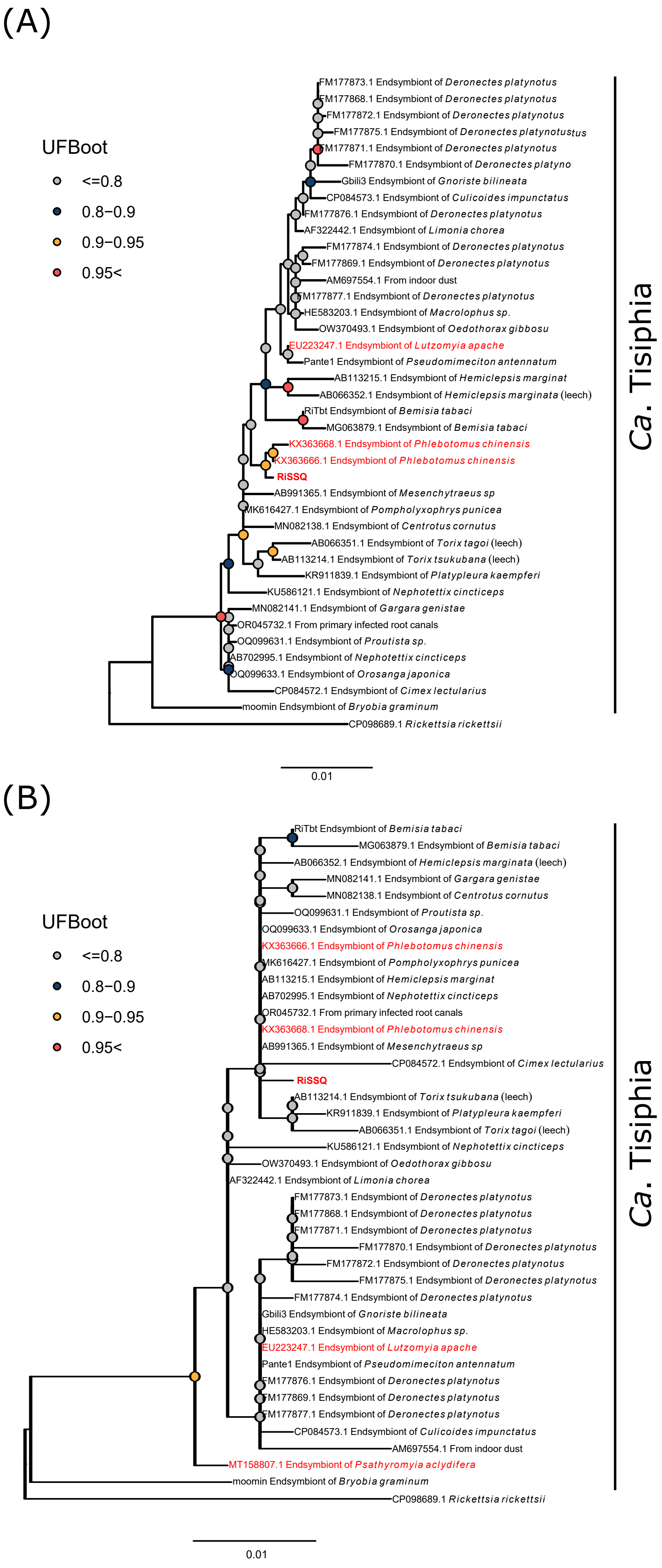

Figure S2 Maximum likelihood (ML) tree for long (A) and short (B) 16S ribosomal RNA sequences (Table S4). UFBoot support above 80% value are described on each node. Labels of taxa detected in Phlebotominae insects are highlighted by red color.

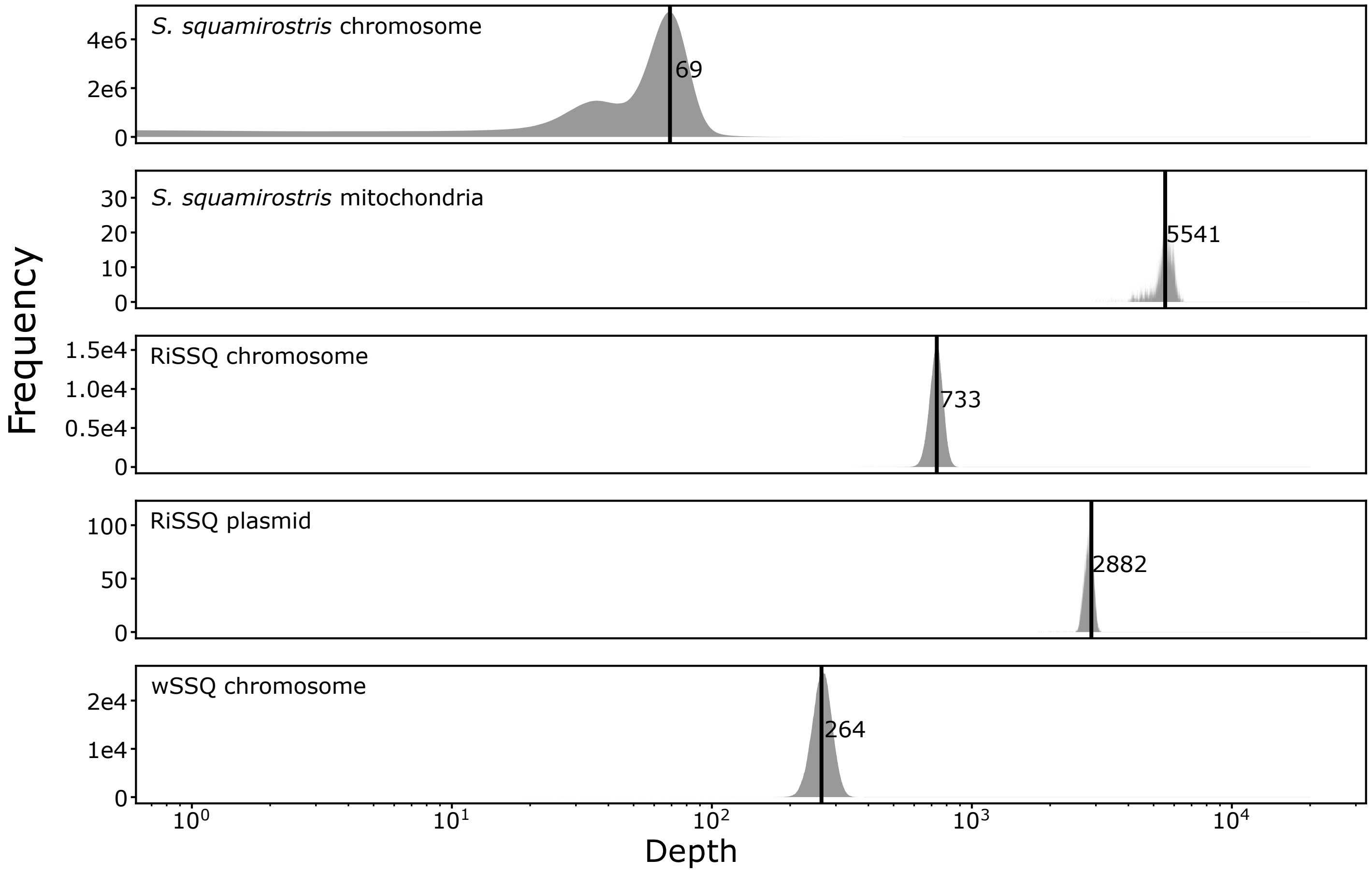

Figure S3 Distribution of read depth (short reads) per nucleotide position on the host chromosome, mitochondria, chromosomes of RiSSQ and wSSQ and the plasmid of RiSSQ. Red lines and the associated numbers indicate the modal points and their values.

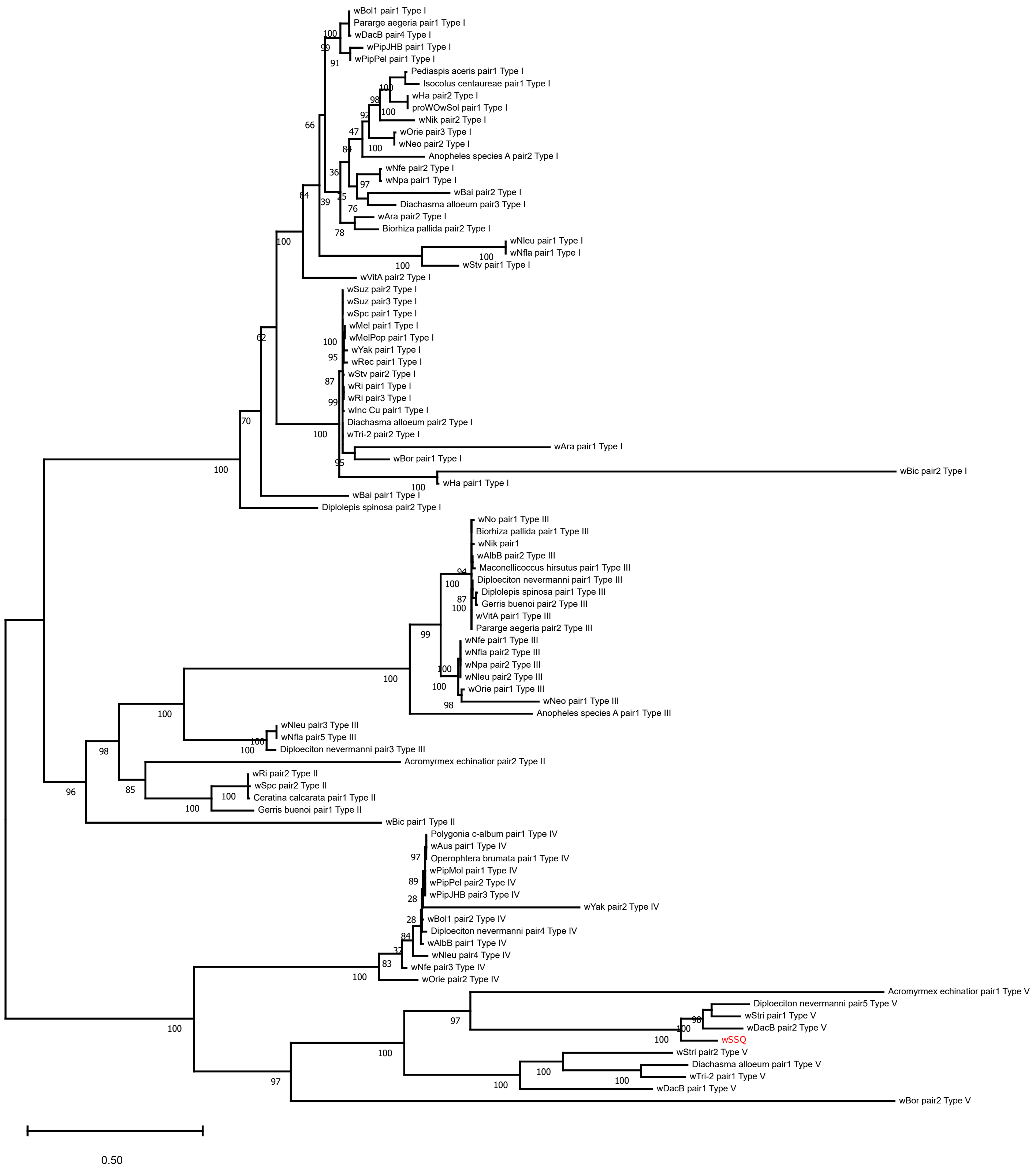

Figure S4 Phylogeny of concatenated cifA/B proteins. The root of the tree was arbitrarily chosen at the middle point.

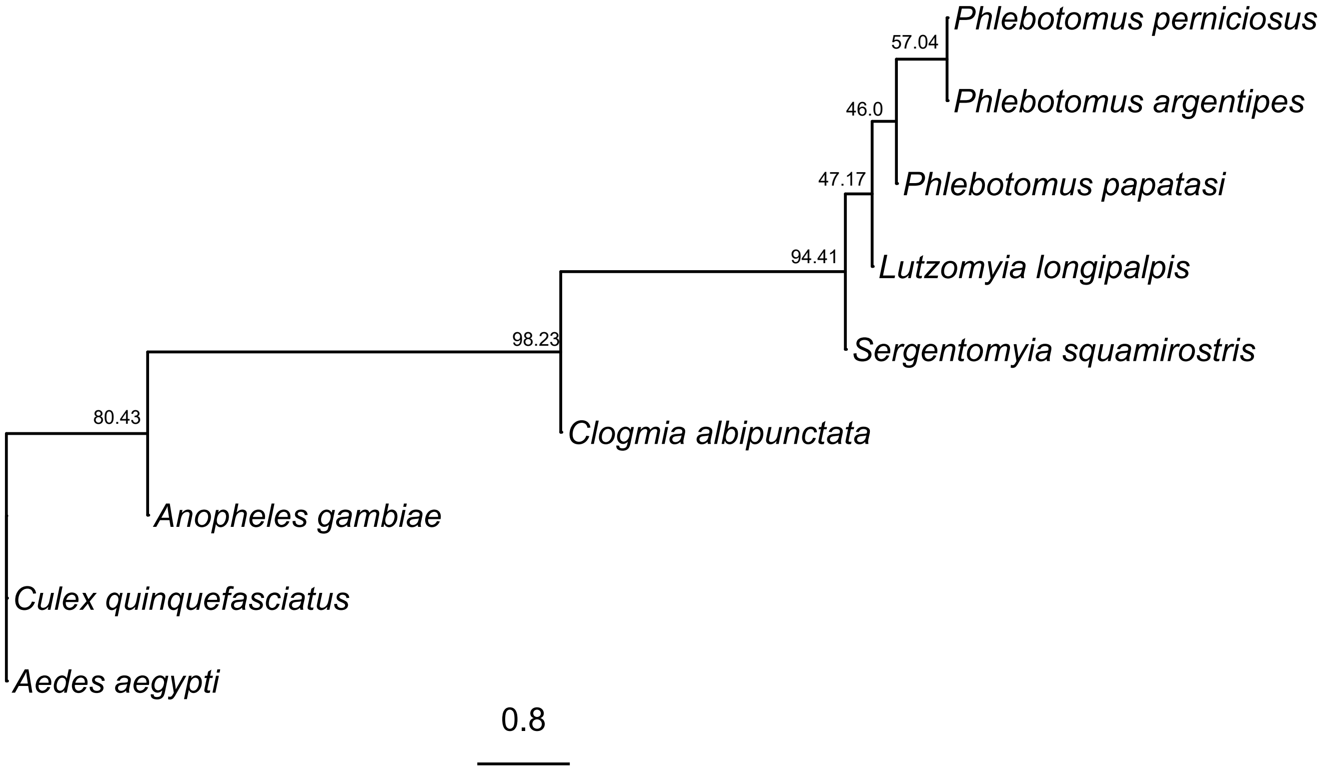

Figure S5 ASTRAL coalescence species tree derived from 1,004 independent orthologous protein gene trees. Branch lengths represent coalescent units, and labels on internal branches indicate quartet support scores for the given topologies. Terminal branch lengths are arbitrary and included solely for visual purposes.
